# Cutoffs and k-mers: implications from a transcriptome study in allopolyploid plants

**DOI:** 10.1186/1471-2164-13-92

**Published:** 2012-03-14

**Authors:** Nicole Gruenheit, Oliver Deusch, Christian Esser, Matthias Becker, Claudia Voelckel, Peter Lockhart

**Affiliations:** 1 Institute of Molecular Biosciences, Massey University, Palmerston North, New Zealand; 2 Institute for Computer Science, Heinrich-Heine-University, 40225 Düsseldorf, Germany

**Keywords:** EST library, mRNA-seq, Transcriptome assembly, *Pachycladon*, Allopolyploidie

## Abstract

**Background:**

Transcriptome analysis is increasingly being used to study the evolutionary origins and ecology of non-model plants. One issue for both transcriptome assembly and differential gene expression analyses is the common occurrence in plants of hybridisation and whole genome duplication (WGD) and hybridization resulting in allopolyploidy. The divergence of duplicated genes following WGD creates near identical homeologues that can be problematic for *de novo *assembly and also reference based assembly protocols that use short reads (35 - 100 bp).

**Results:**

Here we report a successful strategy for the assembly of two transcriptomes made using 75 bp Illumina reads from *Pachycladon fastigiatum *and *Pachycladon cheesemanii*. Both are allopolyploid plant species (2n = 20) that originated in the New Zealand Alps about 0.8 million years ago. In a systematic analysis of 19 different coverage cutoffs and 20 different k-mer sizes we showed that i) none of the genes could be assembled across all of the parameter space ii) assembly of each gene required an optimal set of parameter values and iii) these parameter values could be explained in part by different gene expression levels and different degrees of similarity between genes.

**Conclusions:**

To obtain optimal transcriptome assemblies for allopolyploid plants, k-mer size and k-mer coverage need to be considered simultaneously across a broad parameter space. This is important for assembling a maximum number of full length ESTs and for avoiding chimeric assemblies of homeologous and paralogous gene copies.

## Background

Whole genome duplication (WGD) associated with autopolyploidy and allopolyploidy has been a recurrent and prevalent phenomenon in plant evolution, linked to species diversification and species radiation [1-3]. Difficulty in studying the genomic complexity that WGD entails has slowed progress in understanding the genetic basis of adaptation and speciation in non-model systems [4]. Recently, with the advent of high throughput sequencing, many researchers have turned to analyses of transcriptomes to advance knowledge of evolutionary relationships, and to identify traits and candidate genes potentially important in adaptive diversification. One current international initiative seeks to assemble the transcriptomes of 1000 plant species (http://www.onekp.com/).

Transcriptome assembly has "many informatics challenges" [5] including markedly different expression levels of genes and homeologues, as well as potentially high levels of sequence similarity between duplicated gene copies (for homeologues and other paralogues). Use of a reference transcriptome can facilitate the assembly [6-9]. However, where gene duplication has generated novel sequences compared to the reference transcriptome, these sequences can remain undiscovered or even be assembled as a chimeric sequence with their paralogues. For many plant species a close reference does not exist at all, which makes the assembly even more challenging. In these cases, an assessment of optimal assembly parameters is needed to generate full length ESTs and avoid the production of chimeric sequences formed between homeologous copies, recently duplicated, and very similar genes.

Several pipelines for the assembly of transcriptomes have been suggested in a number of studies. These studies have identified k-mer size as an important parameter. During contig assembly assemblers such as ABySS, Trans-ABySS and Trinity, k-mer size specifies the length of an oligonucleotide sequence used for building the *de Bruijn *graph (see [10] for a short introduction). One k-mer represents a node in the graph while overlaps between k-mers of length k-1 represent the edges that connect the nodes. A contig is assembled by following the connected nodes and edges through the graph. Thus, the length of the chosen k-mer influences the connectivity between the nodes and can affect the result of the assembly considerably. In the case of a transcriptome assembly genes with a low expression level are represented by very few reads with small overlaps in the dataset and therefore can only be assembled with small k-mer sizes. The choice of the k-mer size can either lead to fully assembled or heavily fragmented transcripts, changing the quality of an assembly dramatically. The latter is typically evaluated using metrics such as the length of the longest contig or the N50 length (If the contigs in an assembly are ordered according to their length, the N50 value is the length of the smallest contig, such that the combined length of contigs this size and larger contains at least 50% of the assembled bases. [11]). K-mer sizes between 21 and 47 have been reported as optimal in a number of studies on transcriptome assembly. In some studies only one k-mer size has been used to obtain a 'best' assembly, as assessed by various parameters describing the amount and length of the assembled contigs [12-18]. In other studies, the contigs obtained from assemblies made with a range of k-mer sizes have been reported, since it has been observed that different genes sometimes require different k-mer sizes for optimal assembly [19,20]. Very few studies have at this point reported optimal values or the significance of k-mer coverage [12,15,21,22].

Here we report a successful strategy for the assembly of two reference transcriptomes in *Pachycladon *(*P*. *fastigiatum *and *P*. *cheesemanii*) - an allopolyploid plant genus which has diversified into ten species in the New Zealand Alps within the last 0.8 million years and originated from a hybridization event [23]. The two ancestral lineages represented in each of the extant allopolyploid species are estimated as having diverged from each other within the *Arabidopsis *clade approximately eight million years ago [22,24]. This evolutionary scenario has led to a situation where in *Pachycladon *(i) ortholgues are highly similar between species (> 98% similarity) and (ii) the homeologous genes within each species show less, but still very high, sequence identity (85% - 95%). Thus, the assembly of their transcriptomes is not only complicated by their size (almost double the size of the transcriptome of a diploid organism) but also by the high sequence identity between homeologous copies. This similarity complicates the *de Bruijn *graph considerably. For example, if there are two highly similar homeologues in the transcriptome they will share nodes (k-mers) in the graph while nodes belonging to either sequence will still be connected to the nodes of the respective other sequence. When encountering structures like this in the graph, assembly algorithms tend to terminate the assembly in order to not generate hybrid sequences. This results in rather fragmented assemblies. Using longer k-mer sizes helps to avoid this problem by minimizing the number of connected nodes in the *de Bruijn *graph. However, long k-mer sizes cannot be used to assemble genes with a low expression level as there can be too few overlapping k-mers. Obtaining full length transcripts requires consideration for both k-mer size and k-mer coverage. In the present study we simultaneously evaluate k-mer size and coverage cutoff in generating optimal assemblies for two *Pachycladon *transcriptomes using ABySS [25,26]. We discuss criteria for evaluating our assemblies and also discuss the effectiveness of two currently used transcriptome assemblers Trinity [27] and Trans-ABySS [28].

## Results

### Quality assessment of the reads and *de novo* assembly

Two lanes of paired-end and one lane of single-end Illumina 75 base pair sequences were generated for *P*. *fastigiatum *and one lane of single-end 75 base pair sequences for *P*. *cheesemanii*. Before the 75 nucleotide reads were assembled they were quality checked and trimmed. Each lane was analyzed separately. For both lanes of the paired-end data, there was a significant decrease in quality (where the mean probability of error exceeded 10%) after approximately 45 nucleotides. In both lanes of single-end reads the same quality decrease was reached after approximately 60 nucleotides. All 75,175,754 reads of *P*. *fastigiatum *and 19,191,203 reads of *P*. *cheesemanii *were trimmed to retain the longest contiguous read segment where all nucleotides had a Phred quality score above the cutoff of 20, which is equivalent to one base call error every 100 nucleotides. After this step, only reads longer than 30 nucleotides were used for the assembly. Due to the relatively low quality of the *P*. *fastigiatum *paired-end data only 881 of the 4,029 Megabases could be assembled as paired-end data because reads were only considered as being paired if the length of both reads exceeded 30 nucleotides. If only one of the reads exceeded the length cutoff (618 Mbp) it was added to the set of the single-end reads (1,285 Mbp). After this filtering step 881 Mbp paired-end and 1903 Mbp single-end reads were used to assemble contigs for *P*. *fastigiatum *as well as 1,143 Mbp single-end reads for *P*. *cheesemanii*. The reads for both species were assembled separately using 19 different coverage cutoffs between two and 20 with ABySS v. 1.2.5 [25,26]. 20 different k-mer sizes between 25 and 63 were also considered, resulting in 380 assemblies per species.

### Assessing the assemblies

For each of the 380 assemblies the number and length of the contigs was assessed. In total 23,668,704 contigs were assembled for *P*. *fastigiatum *and 12,264,278 for *P*. *cheesemanii *(see Additional file 1: Table S1). The lowest number of contigs was obtained using a k-mer size of 63 and a coverage cutoff of 20 (3,022 (*P*. *fastigiatum*) and 1,772 (*P*. *cheesemanii*)) while the highest number of contigs was obtained using k-mer size 33 and coverage cutoff 2 (231,969 and 158,290). The percentage of contigs per assembly that were longer than 500 bp varied according to the parameters used. Overall the percentage was higher when large k-mer sizes were used. While the percentage of longer contigs for assemblies made with the same coverage cutoff did not differ significantly when using small cutoffs (2-6), it did vary considerably between different k-mer sizes using higher cutoffs (3% - 20% with a cutoff of 20).

We also compared the total number of assembled bases for each assembly. The highest number of assembled bases for *P*. *fastigiatum *was 46 Mbp (37; 2) while the lowest number was 1.2 Mbp (63; 20). When only contigs longer than 500 bp were considered those numbers dropped to 8.3 (43; 4) and 0.6 Mbp (63; 20). For *P*. *cheesemanii *a maximum of 32 Mbp were assembled using parameters 35 and 2 when all sequences were considered and 5.4 Mbp (43; 4) using sequences longer than 500 bp. The minimal values 0.7 and 0.4 Mbp were found with parameters 63 and 20 for all sequences and sequences longer than 500 bp, respectively.

In order to determine the percentage of reads included in each assembly we mapped the reads of each species against the respective contigs of each assembly. In *P*. *fastigiatum *the maximum percentage of reads mapping to the contigs was 56.07% with parameters 2 and 51, while only 22.51% of the reads mapped with parameters 2 and 25. In *P*. *cheesemanii *the maximum percentage of reads mapping was 55.93% with parameters 3 and 53. The Pearson correlation coefficients between the coverage cutoff or the k-mer size and the percentages of reads mapping were too small to infer a linear correlation (-0.5, -0.45; 0.49, 0.70). However, in both species the highest percentages were associated with low coverage cutoffs (3, 4) and large k-mer sizes (51-55) while the lowest were computed with small k-mer sizes (25, 27).

For each combination of assembly parameter values the length of the longest sequence was determined and annotated against homologues in *A*. *thaliana*. For example, the longest sequences in the *P*. *fastigiatum *libraries (10,134 bases, 10,127 bases and 10,229 bases) were assembled using coverage cutoffs three to five and k-mer sizes 25 to 29 while the shortest sequences (< 3,205) were assembled using coverage cutoffs two to seven and k-mer sizes 57 and 63 (see Additional file 2: Table S2 for details). The longest sequences generated in different assemblies were homologues to different genes. In 105 assemblies the longest sequence generated was homologous to *A*. *thaliana *AT5G40450, an uncharacterized gene whose length is 8,670 bp. In 99 other assemblies, the longest sequence was homologous to GLU1 (AT5G04140; 4,947; [29]). In total, 22 different genes were found in the set of the longest sequences, six of which occurred only once. For only ten of those genes the complete coding sequence was assembled.

Additionally, the N50 and N90 lengths were computed for each assembly of *P*. *fastigiatum *reads. The highest N50 length was 491 for the assembly made with cutoff 20 and k-mer 59, while the smallest N50 length (94) was computed for the assembly with cutoff 2 and k-mer size 25. Overall the N50 length was higher when higher coverage cutoffs and k-mer sizes were used (see Additional file 2: Table S2). The largest N90 length was 149 (cutoff: 19; k-mer: 61) while the smallest was 30 (cutoff: 2; k-mer: 25). The N90 length again was longer when higher coverage cutoffs and k-mer sizes were used. These N50 and N90 values are significantly smaller than the N50 and N90 values for the reference libraries of *A*. *thaliana *(1,548 and 675) and *A*. *lyrata *(1,458 and 561).

### The importance of the k-mer size and the coverage cutoff for transcript assembly

**Figure 1:**
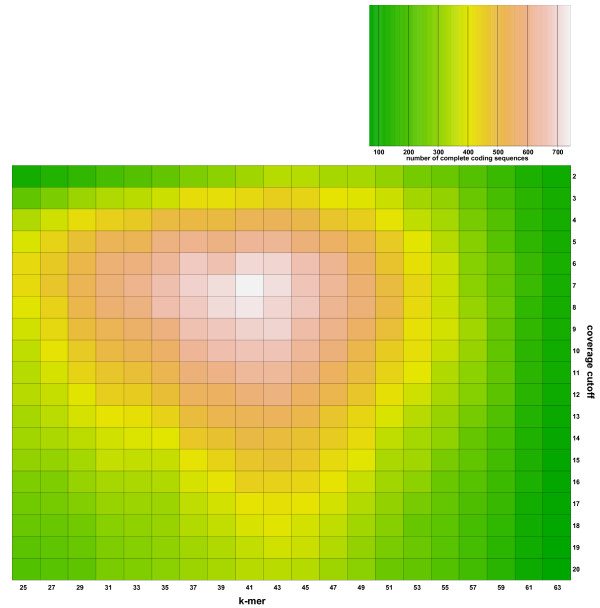
**Number of complete transcripts identified in different assemblies of *P. fastigiatum *reads**. 380 different assemblies were made using ABySS [25,26] and a combination of (i) coverage cutoffs between 2 and 20 and (ii) k-mer sizes between 25 and 63. Transcripts covering the complete coding sequence of the homologue from *A*. *lyrata *or *A*. *thaliana*, respectively, were identified and counted. The maximum number (741) of complete transcripts was identified for coverage cutoff seven and k-mer size 41 while the lowest (70) number of complete transcripts was identified for coverage cutoff 19 and k-mer size 63.

Figure 1 and Additional file 3: Figure S1 show the number of complete coding sequences found in any assembly for *P*. *fastigiatum *(3,912, see Additional file 4: S1) and *P*. *cheesemanii *(2,442; see Additional file 5: S2), respectively. For *P*. *fastigiatum *the highest number of complete sequences (741) was found in the assemblies conducted with k-mer 41 and with coverage cutoff seven while the lowest number (70) was found using k-mer 63 and coverage cutoff 19. For *P*. *cheesemanii *these values differ slightly as the maximum number of complete coding sequences (558) was found using k-mer 41 and coverage cutoff five while the lowest number (58) was again found in the assembly conducted with coverage cutoff 19 and k-mer 63.

**Figure 2:**
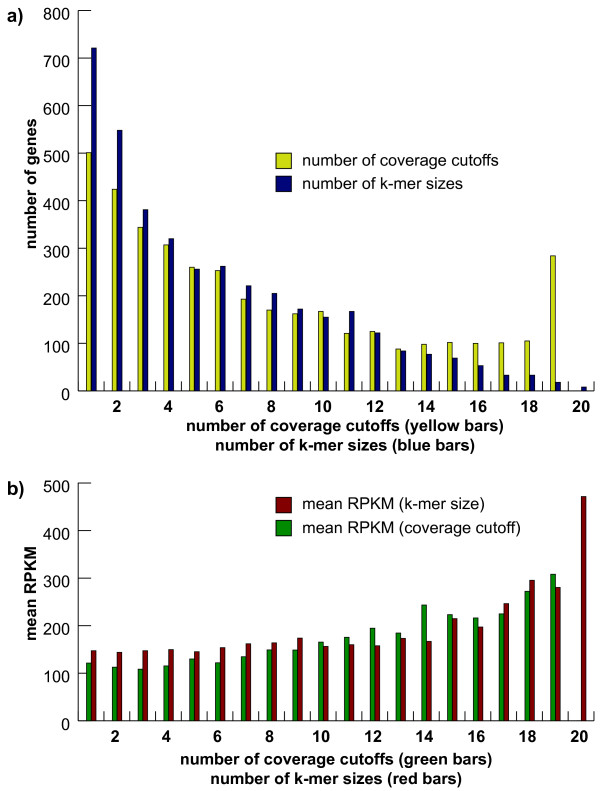
**Number of genes assembled with a certain number of k-mer sizes and coverage cutoffs**. **a)** For each of the 3,912 complete transcripts in the *P*. *fastigiatum *library the number of k-mer sizes (blue bars) and coverage cutoffs (yellow bars) leading to complete transcripts were counted. 721 genes were only assembled using one k-mer size and 501 using one coverage cutoff. **b) **Mean expression levels for genes assembled with a certain number of k-mer sizes and coverage cutoffs. For each set of genes that were completely assembled with the same number of k-mer sizes (red bars) and coverage cutoffs (green bars) a mean expression level was derived by computing the mean of the RPKM values (reads per kilobases and million mapped reads [30]). High positive Pearson correlation coefficients were found between the mean expression level and the number of k-mer sizes and coverage cutoffs.

With *P*. *fastigiatum *none of the genes could be assembled completely with all 380 combinations of the assembly parameters. While there were 284 sequences that were assembled with all 19 different coverage cutoffs, there were only eight sequences that were assembled with all 20 different k-mer sizes (see Figure 2). 501 sequences were complete only in assemblies that used one coverage cutoff. 721 sequences were complete only in assemblies that used one k-mer size. 392 of these sequences were assembled using exactly one parameter combination. Similarly, for *P*. *cheesemanii *the success of gene assembly varied greatly with chosen parameter values. 173 genes were assembled with all 19 coverage cutoffs but only 18 with all 20 k-mer sizes. 445 genes were only completely assembled with one coverage cutoff and 495 genes were only completely assembled with one k-mer. 284 of these genes were assembled with exactly one parameter combination.

### Comparing assemblies in terms of the number of complete transcripts

**Figure 3:**
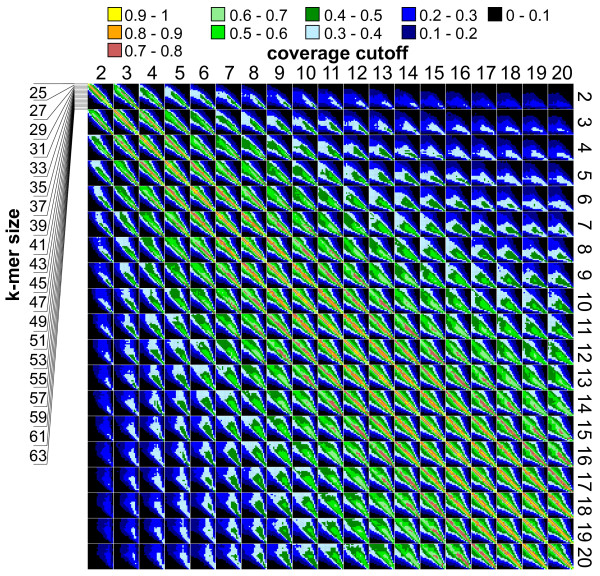
**Amount of overlap between complete transcripts identified in different assemblies of *P*. *fastigiatum *reads**. 380 different assemblies were conducted using ABySS [25,26] and a combination of (i) coverage cutoffs between 2 and 20 and (ii) k-mer sizes between 25 and 63. Transcripts covering a complete coding sequence of the homologue from *A*. *lyrata *or *A*. *thaliana *were identified and counted. These were searched against each other and the number of sequences present in each of two assemblies was determined. The number of overlapping sequences was then divided by the sum of the number of sequences identified in both assemblies and again divided by 0.5 to obtain values between 0 and 1. These percentages were colour coded with yellow representing high values and darker colours low values. A black box means no overlap between the sets of complete transcripts.

To quantify the similarity of assemblies made using different parameter values we counted the number of complete transcripts in each assembly and made pair wise comparisons of assemblies. For each comparison we divided the number of complete transcripts common to both assemblies by the total number of complete transcripts summed across both assemblies. The highest value therefore was 0.5 for perfect overlap and the lowest value was 0 if no sequence was identical between the complete sequences of the two assemblies. These values were then divided by 0.5 to regain easily comparable percentages (see Figure 3). No perfect overlap could be detected between any two assemblies. The highest values were computed for assemblies conducted with near identical k-mer sizes. For example, of the 237 complete sequences found with coverage cutoff 2 and k-mer sizes 25 and 27, respectively, 79 were found in both datasets, which corresponds to an overlap of 67%. Values for the overlap between assemblies conducted with adjacent parameters varied between 67 and 80%. The more difference there was between the assembly parameters the less overlap was detected between the completely assembled sequences. While there was still about 60% overlap if the k-mer sizes differed by four, this decreased to 40 to 50% when k-mer sizes differed by six and to 30 to 40% when they differed by eight. There was no overlap between the 106 and 97 sequences found with parameters 2, 25 and 2, 63. Assemblies conducted with the same k-mer size but different coverage cutoffs showed even less overlap. Between the assemblies made with parameters 2, 25 and 3, 25 only 50% of the sequences were identical. This decreased to 32% with coverage cutoff 4 and further to 1.2% with coverage cutoff 20 (see Figure 3).

### Comparison to trinity assembly

The *P*. *cheesemanii *reads were also assembled using Trinity (k-mer size 25) resulting in 73,641 contigs of which 3,266 were longer than 1,000 bp while most of the contigs (57,012) were between 100 and 200 bp long. The N50 and N90 values of this assembly were 453 bp and 227 bp, respectively. The total number of assembled bases of 30 Mbp was a bit smaller than the maximum value obtained with any ABySS assembly. When only sequences longer than 500 bp were considered the Trinity assembly contained considerably more nucleotides (13.5 Mbp as opposed to 5.4 Mbp). The percentage of reads included in the assembly was 51.78%, which is slightly smaller than the highest value of the ABySS assemblies (55.93%). The longest sequence was 8,179 bp and identified as the homologue to AT1G64790 while the longest sequence in the ABySS assemblies was 8,137 bp. AT1G64790 was also identified to be the longest sequence in 43 ABySS assemblies. 676 contigs in the Trinity assembly represented complete coding sequences while the maximum number of complete sequences identified in any ABySS assembly was 558. After combining the ABySS assemblies 2,442 complete transcripts were obtained. 3,700 sequences in the Trinity assembly spanned more than 55% of an *Arabidopsis *reference gene, which was again less than the 6,448 sequences obtained with all ABySS assemblies.

### Most similar homologues

All ABySS contigs of *P*. *fastigiatum *longer than 100 bp were searched against all plant protein sequences in the nr database using BLASTx [31]. Applying an identity cutoff of 70% the highest percentage of contigs per assembly that had a significant match to the database was 89% with coverage cutoff 20 and k-mer size 51. This percentage was again highly variable between the assemblies. The minimum value was 67.5% for the assembly made with coverage cutoff 2 and k-mer size 25 leaving 65,358 contigs without a hit in the plant nr database. No correlation (Pearson) was detected between the k-mer size or the coverage cutoff and the percentage of contigs with hits in the plant database. A homologous sequence was found in the nr database for 19,494,709 (82%) of the 23,668,704 contigs.

Sequences of *A*. *thaliana *(6,188,006) and *A*. *lyrata *(11,591,097) were found most often as best hits to the *Pachycladon *contigs. Sequences of other species in the *Brassicaceae *lineage were also found as best BLAST hits: For 16,199 sequences the best hit was found with *Boechera divaricarpa*, for 238,304 sequences it was found to be with different species of *Brassica*, and for 589,452 sequences with *Thelungiella halophila*. A small proportion of the sequences had best hits outside of the *Brassicaceae *lineage, e.g. for 92,614 contigs the best hit was found with *Vitis vinifera*, for 68,934 with *Ricinus communis*, and for 60,619 with *Populus trichocarpa*. A small number of the contigs had best hits to algae: 2,873 contigs to *Volvox carteri *and 1,390 to *Micromonas pusilla *CCMP1545. For most of these contigs, homologues in the *Arabidopsis *lineage did exist but were less similar to the *Pachycladon *contigs than the algal sequences.

The lengths of the contigs with hits in the plant database were determined as well as the lengths of the contigs without those hits. Both length distributions were then compared using a Wilcoxon rank sum test. The length of the contigs with hits was significantly longer than the ones for the other sequence set (p < 2.2e-16). The mean length of the contigs with hits was 252 while it was 199 for the other sequence set. The longest sequence in the first set is 10,134 bp while the longest sequence in the other set was 8,292. For both datasets most sequences were between 100 and 300 bp long (77.8%, 86.8%) but the percentage of sequences longer than 1000 bp was slightly higher in the dataset with hits in the plant database (2.2% vs 0.8%). The 776 sequences longer than 3000 bp without hits in the plant database were analyzed further as it is highly unlikely that sequences of this length are comprised of 'nonsense' assemblies. These sequences were found in all assemblies with a k-mer size smaller that 59. When compared against the nucleotide database at NCBI they either hit hypothetical or uncharacterized proteins and genomic sequences. The sequence identity of these hits was mostly below 70%. The longest sequence (8,292 bp; coverage cutoff: 8; k-mer size: 43) did have a hit in the plant database (AT5G40450; uncharacterized protein) but a large number of indels in the alignment reduced the identity to 53%. Interestingly, this sequence passed the filters when searched against the coding sequences of *A*. *thaliana *using BLASTn.

### A comparison of orthologues, paralogues and homeologues

We used two reference transcriptomes for the identification and annotation of homologous transcripts within and between our *P*. *fastigiatum *and *P*. *cheesemanii *libraries. While the *A*. *thaliana *transcriptome is the best annotated reference available, the *Pachycladon *contigs showed the highest identity to the *A*. *lyrata *transcripts. Thus, using only one of the databases as a reference could result in sequences not being annotated either because they were too different to the *A*. *thaliana *sequences or because the *A*. *lyrata *sequences were not annotated. Hence, our contigs were searched against a combined library. Sequences either had a hit in both *Arabidopsis *species or a hit in only one species. All sequences, that covered a minimum length of at least 55% of any *Arabidopsis *reference sequence, were added to the EST libraries. This minimum length ensured that there was at least 5% overlap between orthologues and homeologues in the two libraries. If there were two different overlapping contigs that were homologous to the same *Arabidopsis *gene, these were annotated as possible homeologues. Contigs that were assigned to a specific gene and copy were assembled further using the overlap assembler CAP3 [32]. Using these criteria, we assembled ESTs for 13,284 and 8,890 unique genes for *P*. *fastigiatum *and *P*. *cheesemanii*, respectively. Of these, 5,684 genes were common to both species. All sequences were annotated using Blastn and the combined database of *A*. *thaliana *and *A*. *lyrata *coding sequences (see Additional file 4: S1 and Additional file 5: S2 for a list of annotated genes).

**Figure 4:**
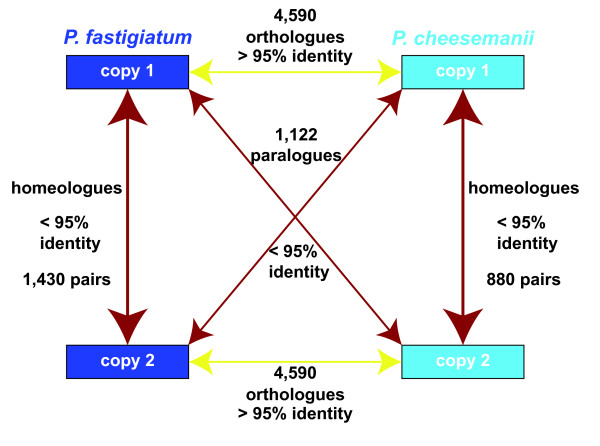
**Schematic of homologous sequences within and between *P. fastigiatum *and *P*. *cheesemanii***. Homologous sequences between *P*. *fastigiatum *and *P*. *cheesemanii *were considered orthologues (4,590 genes) if they were more than 95% identical and paralogues (1,122 genes) elsewise. Homeologues within a species were also less than 95% identical (1,430 and 880 gene pairs).

We counted the number of homeologous pairs present in both species. 547 homeologous pairs were identified as common to both. The mean sequence identity of these homeologous copies was 98.66%, while the minimum was 95.05%. We used this observed distribution of identities to determine the relationship among other homologues occurring in the two libraries. We inferred sequences to be putative orthologues (that is, copy one in both species) if they had greater than 95% sequence identity, and inferred sequences to be likely paralogues (that is, copy one in one species and copy two in the other) if they had less than 95% identity (see Figure 4). The number of genes common to both libraries inferred to have an orthologous relationship was 4,590. This contrasted with 7,600 genes that were only found in the library of *P*. *fastigiatum *and 3,206 genes that were only found in the library of *P*. *cheesemanii*. These genes were either not expressed in the other species or present in contigs that covered less than 55% of the reference gene. 883 gene pairs of the 7,600 genes only found in the *P*. *fastigiatum *library were inferred to be homeologues, while 333 homeologous pairs were identified among the 3,206 genes exclusive in the *P*. *cheesemanii*. In total 1,430 (547 common and 883 only) homeologous pairs were assembled for *P*. *fastigiatum *and 880 for *P*. *cheesemanii *(547 common and 333 only). The total number of *Pachycladon *genes represented by these sequences was 16,460.

In order to determine if the 3,206 genes only found in the library of *P*. *cheesemanii *were a result of differential expression between the species we mapped the reads of *P*. *fastigiatum *and *P*. *cheesemanii *against these genes using Bowtie v. 0.12.5. [33] and allowing for up to three mismatches. We then compared the number of reads mapping to each of the genes in both species. This was done after normalizing for the total number of reads mapping in each species. For nine of the sequences no expression was determined in *P*. *fastigiatum*. 1,689 genes had a higher RPKM value in *P*. *fastigiatum *than in *P*. *cheesemanii *while 1,517 had a higher expression level in *P*. *cheesemanii*. A paired Wilcoxon rank sum test was conducted to determine if the expression levels were significantly higher. In both cases the difference in expression level was highly significant (*p *< 2.2e-16) indicating that the coverage of those genes was too low for an assembly of at least 55% in one species. A comparison of the genes present only in either one of the libraries showed that for 1,122 of these genes the respective other copy was assembled in the other library. This illustrates a relatively high degree of differential expression between the two homeologous copies.

The sequences present in both libraries (547 homeologous pairs, 4,590 paralogues) were compared to homologues from *A*. *lyrata *and *A*. *thaliana*. The mean identity of sequences from both *Pachycladon *species with respect to sequences of *A*. *thaliana *was found to be approximately 92%, while the identity to the *A*. *lyrata *sequences was slightly higher. The minimal identity of the BLAST alignments between the homologues was 62% and the maximum 100%. To determine if it was possible to assign gene copies in the *Pachycladon *species to maternal and paternal ancestral lineages, a similarity comparison was made between sequences for the 547 homeologous gene pairs with *A*. *lyrata *and *A*. *thaliana*. However, no set of genes was identified that showed a significantly higher identity to the reference genes consistent with the suggestion that the homeologous genes both stem from the maternal ancestral lineage. Thus, we were unable to unambiguously map gene copies to distinct evolutionary lineages.

### Observations on specific genes that highlight issues for assembly

We investigated the number of reads mapping to seven genes that were expressed at different levels and exhibited different degrees of sequence similarity. This was done allowing for no mismatches and also up to 3 mismatches per read. The motivation for allowing mismatches was to accommodate potential sequencing errors that might occur with high density of reads and also to demonstrate the assembly problem caused by having very similar homeologous sequences in the dataset.

**Figure 5:**
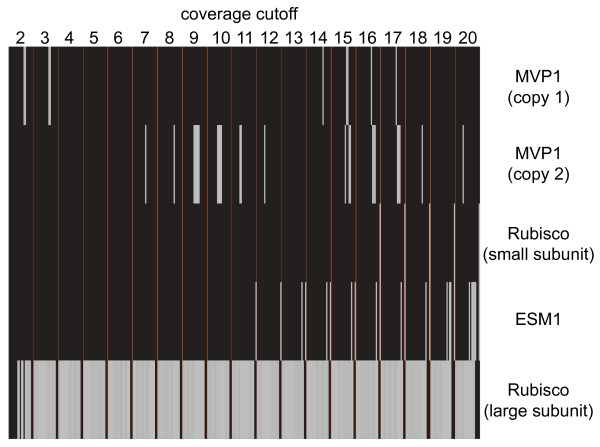
**Assembly patterns in the *P*. *fastigiatum *library for five example transcripts**. All 380 assemblies (coverage cutoffs 2 to 20 and k-mer sizes 25 to 63) were screened for complete transcripts of five example genes (AT1G67090, AT3G14210, two homeologous sequences of AT1G54030, and ATCG00490). If a complete transcript was present in an assembly it was marked in grey and in black otherwise.

Two genes studied (rbcS, ESM1) had a very high expression level. For these a complete transcript was assembled under very few k-mer size and coverage cutoff combinations. Homeologous copies were not assembled for either gene. In the *A*. *thaliana *genome, four genes encode the small subunit of Rubisco [34]. Of these four genes only one (AT1G67090) was assembled completely in five different assemblies using coverage cutoffs 16 to 20 and k-mer 63. For the other three genes, only contigs that spanned less than 55% of the reference sequences were found. Another interesting case concerned the contigs for the homologues to MVP1 (AT1G54030), a myrosinase-associated protein [35]. One MVP1 gene copy was assembled using 25 different parameter combinations with coverage cutoffs seven to twelve, 15 to 18, and 20 and k-mer sizes between 37 and 55 while a second MVP1 gene copy was assembled using nine different combinations using cutoffs two and three and 14 to 17 but only using k-mer sizes 49 and 51. A third MVP1 gene copy could also be assembled by combining smaller contigs using CAP3. Comparison to the transcriptome of *A*. *lyrata *revealed a duplication of MVP1 on chromosome 3 explaining the occurrence of the third copy in *P*. *fastigiatum*. Sequence comparison and similarity between *A*. *lyrata *and *Pachycladon *homologues was used to annotate the homeologous gene copies (see Figure 5 for assembly patterns of these examples). The three copies of MVP1 were all highly similar and had a low to medium expression level. Two other genes investigated (ATCG00490 and AT1G75680) had a low expression level and were found to be robust to choice of parameter values in most assemblies. In *P*. *fastigiatum *the homologue to AT1G75680 (glycosyl hydrolase 9B7 [36]) was the gene found in most assemblies (332). Although AT1G75680 is nuclear encoded, only one gene copy was found under different assembly conditions. Not all parameter combinations led to a completely assembled sequence for this gene, but there was at least one partial sequence from each of the 19 coverage cutoffs and 20 k-mer sizes. The gene found with the second highest number of assemblies was the chloroplast located large subunit of Rubisco (ATCG00490; 329; [34]) for which only one copy was present in the transcriptome. Again there was at least one complete sequence for each of the coverage cutoffs but only k-mer sizes between 25 and 59 led to a completely assembled sequence.

The number of reads mapping to each of these sequences established different expression levels of the corresponding genes. 215,536 reads mapped to the sequence of rbcS and 195,295 to ESM1, only 10,937 reads mapped to one homeologous copy of MVP1, while 1,854 reads mapped to the other homeologous copy. 8,903 reads mapped to the paralogue of MVP1. 3,579 reads mapped to rbcL and 3,420 to the homologue of AT1G75680. When allowing for up to three mismatches, the number of additional reads mapping to the sequences did not scale proportionally. While the number of reads mapping to ESM1, rbcL and the homologue to AT1G75680 increased by about 9%, it increased by 28% for rbcS and 229% for one copy of MVP1. The number of reads mapping to the other two sequences of MVP1 increased by 10% and 14% (see Table 1). In total 4,815 reads mapped to the sequences of the two copies of MVP1 when allowing for up to three mismatches. This number is more than twice the number of reads mapping without mismatches to one of the sequences. Even when no mismatches were allowed 113 (6%) reads mapped to both sequences. While no read was identical between the two copies and the duplicated third sequence when allowing for no mismatches, there were 250 and 244 identical reads when allowing for up to three mismatches, respectively.

**Table 1:** Number of reads mapping to seven example transcripts.

sequences/# mismatch	0	1	2	3	# assemblies
**AT1G67090 **	215.536	260.217	268.808	276.132	5

**AT3G14210 **	195.295	209.891	211.720	212.871	24

**AT1G54030 **	1.854	2.607	4.069	6.111	9

**AT1G54030 **	10.937	11.781	12.086	12.521	25

**AT1G54030 **	8.903	9.555	9.672	9.823	0

**ATCG00490 **	3.579	3.848	3.889	3.910	329

**AT1G75680 **	3.420	3.675	3.713	3.739	332

The number of reads from the *P. fastigiatum *dataset mapping to the transcripts of AT1G67090, AT3G14210, three sequences of AT1G54030 (two homeologues and one paralogue), ATCG00490, and AT1G75680 were determined as well as the number of assemblies the complete transcripts of these genes were identified in.

A comparison of assembled MVPI homeologues in the *P*. *fastigiatum *library identified eight different regions of length 35 to 47 bp that were identical between the sequences. The first identical region between the copies is located between nucleotide 93 and 139 of the MVP1 coding region. All contigs that span this region were assembled with k-mer sizes greater than 47 regardless of the coverage cutoff. In assemblies made with smaller k-mer sizes two overlapping contigs were produced for this region, however they were not joined (a maximum overlap of 43 base pairs was observed).

### Separate and joint assembly of reads for specific genes

Reads mapping to assembled ESTs for the seven mentioned genes were removed from the pool of reads. Reads mapping to each of these genes were then assembled separately with k-mer lengths 25 to 63, and without specifying a coverage cutoff. When no coverage cutoff is specified, ABySS determines the coverage cutoff automatically according to the actual k-mer coverage of each respective sequence. The automatically determined coverage cutoffs for individual genes varied greatly. For example, while the determined coverage cutoff for rbcS was between 46.8 for k-mer size 63 and 122.8 for k-mer size 25, it was between 4 and 9.64 for rbcL (see Additional file 6: Table S3 for details). We observed that if reads for different genes were jointly analysed, the automatically determined coverage cutoff always dropped to two. This occurred irrespective of how many and what reads from different genes were assembled.

To determine the effect of including reads in an assembly where there are mismatches to the contig sequence, the reads mapping to each sequence were assembled with k-mer sizes 25 to 63. Four different read datasets were used for each gene sequence: no mismatch, up to one mismatch, up to two mismatches, and up to three mismatches. If one contig were assembled with each k-mer value (2-63) and for 0-3 mismatches, then 20x4 (80) full length identical transcripts are expected for each gene. However, the resulting contigs not only varied in length and number but also in their coverage. After the separate assembly of the seven example genes, we combined the datasets of all seven genes to simulate a small transcriptome assembly.

In the separate assemblies, the genes with high expression levels (ESM1, rbcS) were assembled to a full length transcript in most assemblies. The assembly of ESM1 was the least sensitive to changes in parameter values. With this gene, there was no fragmentation as all 80 assemblies each produced exactly one contig. This was also true for the assemblies of rbcS when the read dataset without mismatches was used. As soon as reads with one to three mismatches were included, some assemblies were fragmented. Full length transcripts were only found with k-mer sizes 61 and 63. The highest fragmentation (eight contigs) was found with k-mer size 27.

In the joint assemblies the results differed dramatically from the separate assemblies. While for ESM1 there was a full length contig for each k-mer when the reads mapping without mismatches were used, 12,930 contigs were assembled when one mismatch was allowed, 15,500 with two mismatches and 16,899 with three mismatches. Most of these sequences were smaller than 120 bp. Some longer contigs (up to 1,103 bp) were obtained in the dataset with one mismatch, though none of those were full length transcripts. The same observation was made for rbcS. While there were 20 contigs for the dataset without mismatches, there were 11,361, 13,636, and 14,287 for the other datasets. No full length transcripts were found for these, with the maximum length transcript being 233.

With the three MVP1 homologues (two homeologues, one paralogue) and the separate assemblies highest fragmentation occurred with small and large k-mer sizes. K-mer sizes between 25 and 51 produced full length contigs for one homeologous copy (except for the first six nucleotides). Increasing the number of mismatches increased the amount of fragmentation and decreased the number of full transcripts obtained. The assembly of the duplicated MVP1 gene sequence showed the least fragmentation of MVP1 homologues.

The results for the joint assemblies were similar. For the first homeologous copy of MVP1 no full length transcript was found. Full length transcripts were found for the second homeologous copy with k-mer sizes 49 to 59 in the dataset without mismatches and 51 to 61 in the others. The lowest degree of fragmentation again was found for the third sequence of MVP1. Full length transcripts were assembled with k-mer sizes 29 to 55. In the other assemblies, the sequence was fragmented into three contigs.

The lowest degree of fragmentation in the separate as well as the joint assemblies was found for the two sequences with a low expression level. Only assemblies with large k-mer sizes, regardless of separate or joint analyses, failed to produce fully assembled sequences. Allowing for mismatches where genes had low expression levels resulted in a decrease of fragmentation in assemblies with high k-mer sizes indicating that the additional reads were crucial for the assembly of regions with low coverage.

### Gene expression levels and assembly parameters

In order to determine whether or not a relationship existed between the expression level of a gene and assembly parameters as previously suggested [37], the trimmed reads were mapped against the sequences of each of the full length transcripts using Bowtie v. 0.12.5 and an expression level (RPKM; [30]; definition given in Materials and Methods section)) was derived.

In *P*. *fastigiatum*, rbcS (AT1G67090) had the highest expression level (49,860) followed by ESM1 (AT3G14210; 19,421; [38]), LTP1 (AT2G38540; 19,106; [39]), the homologue to AT1G72290 (16,323; [24]), and VSP1 (AT5G24780; 10,989; [40]). For each gene the number of coverage cutoffs and k-mer sizes used for assemblies, in which a full transcript was obtained, was determined (see Figure 2). ESM1, for instance, was assembled in 24 of the 380 assemblies. No complete transcript was found in assemblies conducted with coverage cutoffs between two and ten. For each assembly made using cutoffs 11 to 20 one transcript was obtained using k-mer size 63. A complete transcript for ESM1 was also obtained using cutoffs 13 to 20 and k-mer size 57. With cutoff 19 full length transcripts were obtained using k-mer sizes 51 and 55. In addition there were fully assembled transcripts found using cutoff 20 and k-mer sizes 47, 51, 53, 55, 57, and 63. In summary, ESM1 could be assembled using ten different coverage cutoffs and six different k-mer sizes. In contrast, 721 genes were assembled with exactly one k-mer size but with possibly varying coverage cutoffs, while 501 genes were assembled with exactly one coverage cutoff and varying k-mer sizes. Only eight genes were assembled with all 20 k-mer sizes, while 208 genes were assembled with 19 coverage cutoffs, respectively. 392 genes were fully assembled using exactly one combination of these two parameters. Next, the relationship between gene expression level, k-mer size, and coverage cutoff was investigated to determine whether the expression level of a gene affected its assembly over a wide range of assembly parameters. First, the genes were binned into different categories according to the number of k-mer sizes for which a complete transcript was assembled. For example, as mentioned above, the sequence of ESM1 was fully assembled using six different k-mer sizes and was therefore binned in category 6. The number of genes falling in each of the categories is shown in Figure 2a (blue bars). The mean expression level of all genes in each category was determined (Figure 2b, red bars). This was also done for the coverage cutoffs, where 19 different categories were possible (see yellow bars in Figure 2a and green bars in Figure 2b). Finally, a correlation coefficient was computed between the mean expression level of genes in each category and the number of coverage cutoffs or k-mer sizes per category. When the expression levels of all genes were used, no correlation was observed. This situation changed if genes with an extremely high level of expression were excluded from the analysis, a result that might be explained by the observation that a very high expression level can lead to highly fragmented assembly patterns similar to a very low expression level. When 94 genes with an RPKM value greater than 1000 were excluded from the correlation analysis we observed a positive correlation. The Pearson correlation coefficient for the coverage cutoffs (*r *= 0.95) was higher than the correlation coefficient for the k-mer sizes (*r *= 0.82). This means the higher the mean expression level of the genes in a category was, the more different k-mer sizes and coverage cutoffs lead to a full transcript in the assembly.

In *P*. *cheesemanii *the genes with the highest expression levels were LTP4 (AT5G59310; 24,868; [41]), a plant defensin gene (AT5G44430; 18,341; [42]) and photosystem I light harvesting complex gene 3 (AT1G61520; 16,626; [43]). The rbcS and the LTP1 gene were only assembled to 68% and 80% of their respective *Arabidopsis *orthologues, while ESM1 and VSP1 had an RPKM of 9,329 and 1,612. 113 genes had an RPKM value greater than 1000 and these genes were excluded from the correlation analysis. Calculating the Pearson correlation coefficients for this reduced set gave coefficient values of *r *= 0.96 and *r *= 0.84 between RPKM values and coverage cutoffs and k-mer sizes, respectively.

## Discussion

In this study we investigated the difficulty of transcriptome assembly in the case of an allopolyploid transcriptome in which there were high levels of similarity between homeologues. Our findings supported earlier studies which have identified the importance of k-mer size for optimal assembly [28]. That is, while genes with low expression levels are more easily assembled (that is longer contigs can be obtained) with small k-mer sizes, assembly of genes with higher expression require large k-mer sizes. This is particularly true when there is a high degree of similarity between homeologues. Additionally we showed that simultaneously varying k-mer size with the coverage cutoff had a significant impact on the success of gene assemblies. Most importantly we showed that both parameters (k-mer size and k-mer coverage) need to be optimized for each gene or set of genes in the transcriptome depending on their properties. Currently, such extensive evaluation of parameter space is not conducted by transcriptome assemblers such as Trans-ABySS and Trinity, and thus will likely produce suboptimal assemblies with some datasets.

### Comparison of homologues

The parental species of the genus *Pachycladon *diverged about 8 million years ago while the different *Pachycladon *species diverged only 0.8-1.3 million years ago [23]. Therefore we expected greater similarity between orthologues than between the homeologues within each species. The analysis of 547 homeologous genes whose duplicated copies were present in both species confirmed this expectation. While the identity between homeologous genes had a range of 70% to 90%, orthologues were at least 95% identical.

This high degree of similarity between homeologues created a high risk of assembling chimeric sequences, where one part of the sequence derives from one copy while another part derives from the other copy. Furthermore we wished to avoid assigning contigs for different homeologous copies to the wrong copy. For this reason we only evaluated contigs that were assembled to be longer than 55% of the reference gene to which they were annotated. This minimal length ensured a minimum of 5% overlap between *Pachycladon *homeologues. If this overlap was at least 200 bps it could reliably be used to distinguish copies.

Interestingly, only 35% (5,684) of the genes that were unambiguously identified were present in both libraries. Among these, both copies were present for 547 genes, while for 4,590 genes only one copy was identified in both species. For 65% (7,600 and 3,206) of the assembled sequences no counterpart was found in the respective other library although for a surprisingly high number of genes (1,122) the respective second copy was found in either one of the two libraries. This relatively small percentage of overlap between the assembled libraries and greater number of sequences in the *P*. *fastigiatum *transcriptome might have resulted for different reasons. First the number of reads obtained from the *P*. *fastigiatum *transcriptome was almost three times as high as the number of reads from the *P*. *cheesemanii *transcriptome, making it more likely that more genes with a very low expression level would be assembled for *P*. *fastigiatum*. The availability of the paired-end data for *P*. *fastigiatum *also helped to assemble genes where the length of an identical region exceeded 63 bp. This was the case even though these data had low quality than the single-end data and therefore were significantly shortened during the trimming process. Sequences only found in the *P*. *cheesemanii *dataset (3,206) on the other hand has different reasons. Lower expression levels or the lack of expression in *P*. *fastigiatum *lead to several small contigs as the coverage in some regions is not high enough to combine them. The use of the respective other homeologous copy in the other species is another reason, while for some genes higher expression levels in *P*. *fastigiatum *result in fragmented assemblies because of the introduction of sequencing errors. Mappings of the *P*. *cheesemanii *reads against the *P*. *cheesemanii *contigs did not reveal any substantial SNPs that might stem from the three different biological replicates used. Nonetheless it is still possible that for some genes in *P*. *cheesemanii *and *P*. *fastigiatum *alike the assembly would also be complicated because of SNPs between the different accessions.

The comparison of homeologous pairs found in both species with their homologues in *A*. *lyrata *and *A*. *thaliana *confirms the recent finding that both parental species have arisen from the *Arabidopsis *lineage [44]. Since both parental genomes have similar divergence estimates from both *Arabidopsis *species, the presence for example of the duplicated gene of the MVP1 in the *A*. *lyrata *and *Pachycladon *genomes does nevertheless hint at a higher similarity in gene content to *A*. *lyrata*. This suggestion was also supported in evaluations of the best BLAST hit for each contig. Almost twice as many contigs had a gene from *A*. *lyrata *as a best BLAST hit compared to the number of best BLAST hits to *A*. *thaliana*. The recent finding that the parental species of the genus *Pachycladon *both stem from the *Arabidopsis *lineage [44] instead of one parent stemming from the *Arabidopsis *and one from the *Brassica *lineage [23] also received support from the observation of a relatively low number of contigs having best hits to *Brassica *species. The number of contigs found with a best BLAST hit outside of the *Brassicaceae *lineage strengthen the argument that one reference transcriptome might not be sufficient to fully annotate a newly assembled transcriptome where there might occur genes that are no longer present in the reference species.

The mean length of the sequences without a hit in the nr plant database is slightly smaller than the length of sequences with a hit, confirming the observation that the annotation rate for sequences shorter than 200 bp is not as reliable as for longer sequences [45]. Considering the surprisingly high number of longer sequences (> 3000 bp) without a hit in the database, an annotation pipeline containing both a protein and a nucleotide dataset might lead to a higher annotation rate.

### K-mer size and assembly

Comparison of assemblies made with different parameter combinations showed that the k-mer size had a significant impact on the length of contigs as previously observed [22,46]. While contigs assembled with low k-mer sizes tend to be smaller than contigs assembled with higher k-mer sizes, there are also many more contigs assembled with low k-mer sizes. This is largely explained by identical regions in different genes. As soon as the length of an identical region exceeds the length of the chosen k-mer size, the different genes cannot be assembled without risking the formation of chimeric sequences. In this situation, ABySS generates contigs that overlap but it does not combine them without further information. This produces a highly fragmented assembly. To reduce fragmentation, longer reads and higher k-mer sizes can be used. However, genes will not be assembled where there is insufficient overlap between longer k-mers. Genes expressed at low levels can only be assembled using small k-mer sizes. To assemble the largest possible number of contigs with the longest length, a range of k-mer sizes is required. In most studies of EST libraries reported to date the goal is to assemble as many genes as possible, which might explain why low k-mer sizes (typically between 21 and 47), have been used in previous studies [12-18].

### Coverage cutoff and assembly

Although it appears not to have been used to advantage in reported transcriptome studies, the coverage cutoff can also help to avoid assembly problems when there are identical regions between homeologues or paralogues. If one of two highly similar homeologues has a high expression level while the other homeologue has a low expression level, the highly expressed homeologue can be assembled using high coverage cutoffs. Doing this will exclude from assembly the low coverage reads that belong to the second homeologue. Once the highly expressed reads have been assembled (and removed from the pool of reads) then the assembly of reads from the lowly expressed homeologue can be made using a low coverage cutoff and a small k-mer size. Indeed it may make little sense to search for a single set of 'best' assembly parameters for a transcriptome. Such an approach is likely to limit the number of genes that can be assembled in the EST library.

### Trans-ABySS and Trinity

The Trans-ABySS [28] assembler was tested on our datasets because earlier benchmarking analyses showed an improvement in the quality of Trans-ABySS assemblies over ABySS assemblies. Trans-ABySS takes contigs assembled using ABySS with different k-mers as input and then conducts a BLAT [47] search to find nearly identical contigs obtained in these assemblies. Overlapping contigs with identical sequences are then assembled further using CAP3 [32]. This step reduces the number of contigs considerably. For our dataset this method resulted in a high number of hybrid sequences for the homeologous copies. The reason for this is that if the distance between two SNPs between the homeologous copies is greater than the k-mer used, ABySS produces contigs that overlap by exactly the sequence between the two SNPs. CAP3 is an overlap assembler that combines sequences by a majority rule, meaning that contigs stemming from different homeologous copies will be combined randomly if an overlap of identical sequences is present. As no parameter setting of percent identity and length of overlap were available with Trans-ABySS to prevent the assembly of hybrid sequences a more conservative approach to transcriptome assembly was investigated in the present study (results not shown).

Trinity [27] is a transcriptome assembler that does not generate one large *de Bruijn *graph for the whole dataset but first generates linear contigs from seeds (filtered k-mers) in the Inchworm step first. These linear contigs are then converted into *de Bruijn *graphs in the Chrysalis step. This method was tested on the *P*. *cheesemanii *dataset and indeed was able to reduce the effect of different expression levels of the genes. Genes that were known to have a low expression level were assembled with the same parameter as genes with a very high expression level. Nonetheless the N50 and N90 value as well as most other assessment parameters used do not show any significant improvement to the single assemblies conducted with ABySS. Only the amount of bases assembled in the sequences longer than 500 bp indicate that sequences assembled with Trinity are longer than with ABySS. Trinity assembles 78 complete transcripts more than any single ABySS assembly but 1,806 complete transcripts less than were obtained with all ABySS assemblies. Although Trinity is able to accommodate for differences in the expression level, the default k-mer size specified is 25. In our case this means that homeologous that have identical regions of more than 25 nucleotides cannot be assembled anymore. Restricting the k-mer parameter space results in a fragmented assembly. With Trinity, the k-mer can only be increased to a maximum of 32 making this assembler, while promising for diploid organisms, does not significantly improve the transcriptome assembly of allopolyploidy species similar to *Pachycladon*. Further, in the situation where a homeologous gene copy has a very low expression level relative to the other copy, this sequence is filtered out in the Butterfly step as it is assumed to be the result of sequencing errors.

### Assessment of assemblies

Parameter estimates used to assess *de novo *assemblies have previously included the number of the assembled contigs, the length of the longest sequence, and the N50 length. Our study of *Pachycladon *assemblies and also previous studies suggest that all three are related, and the first two parameters can be predicted just from the k-mer size used. Assemblies conducted with small k-mer sizes have more contigs because of the higher fragmentation of the sequences. This fragmentation also leads to a higher number of smaller contigs and therefore to a smaller N50 length. Assemblies conducted with high k-mer sizes produce fewer contigs (and therefore represent less of the transcriptome), a higher percentage of longer contigs and a higher N50 length. The use of the N50 length is most appropriate when assembling whole genomes but when evaluating the assembly of a transcriptome, in which the lengths of the genes are highly variable by default, a high N50 length does not necessarily indicate a higher quality transcriptome assembly. Rather, assemblies that have a high N50 length select against the assembly of shorter genes. This suggests that less significance should be placed on N50 length and more emphasis should be placed on how many and what sequences are assembled. This suggestion is supported by the observation that the longest sequence in each *Pachycladon *assembly was not the same gene. In our 380 assemblies 22 different genes were identified as being the longest transcript. Other parameters like the percentage of reads included in the assembly or the amount of sequences assembled indicate how much of the actual transcriptome is captured in the assembly. Optimal k-mer size and coverage values derived from these parameters favour the use of small coverage cutoffs and larger k-mer sizes. Nonetheless, one of the most important uses of an assembled transcriptome is for differential expression analysis. Particularly when dealing with polyploidy species it is crucial to be able to distinguish the two homeologous copies of one gene in order to distinguish expression levels of both copies. The more fragmented an assembly is, the harder it is to reliably distinguish contigs belonging to either of the two copies. While the amount of data generated and included in the assembly are important parameters, they do not give an indication of how fragmented are the assemblies.

### Assessment should be based on the total number of full length transcripts

While it is obvious that there must be one best assembly with regard to whole genomes [25], with transcriptomes assembly must be optimized for each of the transcripts separately, making that task much more challenging. Instead of assembling only one genome the assembly of a transcriptome is analogous to the simultaneous assembly of several thousand small genomes wherein optimal parameters need to be found for each genome.

In our study, the highest number of full length transcripts was found with k-mer size 41 and coverage cutoff seven for *P*. *fastigiatum *and coverage cutoff five for *P*. *cheesemanii*. The lowest number of full length transcripts was found using k-mer size 63 and high coverage cutoffs. This suggests that many genes shared an optimal or near optimal parameter combination at the mid range of our parameter values. While k-mer size 41 was high enough to distinguish between the homeologous copies it was also small enough to assemble genes with a medium expression level. Coverage cutoffs seven and five were also effective in assembly when genes in our dataset exhibited a medium level of expression. Reducing the coverage cutoff increased the amount of noise and the complexity of the assembly problem, thereby reducing the total number of full length assembled transcripts. Similarly, increasing the coverage cutoff above 10 also significantly reduced the total number of such transcripts, because relatively fewer genes had sufficiently high expression levels. High k-mer sizes also led to suboptimal assemblies. K-mer sizes higher than 41 produced a reduced number of full length assembled transcripts irrespective of coverage cutoffs, a result consistent with most transcriptome assemblies reported to date which typically report optimal k-mer sizes smaller than 41. An important point of note is that the optimal k-mer size and coverage cutoff is expected to vary between organisms and also between different read datasets for the same organism. In respect of the later, our results suggest that the absolute number of reads will influence the optimal k-mer size and coverage cutoff values for each gene in the transcriptome.

Comparison of assemblies revealed a surprising lack of overlap with respect to the full length transcripts. The maximum number of full length transcripts found in one assembly was 741. If only this assembly had been conducted, 3,171 sequences would not have been assembled to full length transcripts. For many genes near identical parameter values gave similar assembly results (60-80% overlap), while more distinct parameter combinations produced assemblies with little overlap. Transcripts found to be full length under one set of assembly conditions often occurred in other assemblies in a more or less fragmented state. Such fragmented sequences are less useful for differential expression analyses as the statistical power is less for smaller sequences [48]. Furthermore in allopolyploid plants it might be difficult to assign reads to the appropriate homeologue under such conditions. These considerations provide further justification for the idea that the best measure of a transcriptome assembly should be the length of the transcripts.

The realization that an optimal assembly requires optimization for each gene becomes even clearer when the parameter combinations for which full transcripts were assembled are considered. There was no gene in our dataset whose assembly was not influenced by either the coverage cutoff or the k-mer size. For example, although there were some genes in *P*. *fastigiatum*, that could be assembled with a wide range of parameter combinations such as glycosyl hydrolase 9B7, many genes did not assemble completely with only one specific coverage cutoff and/or one specific k-mer size (see Figure 2). The analysis of the expression level and similarity between the genes suggests that there are mainly two reasons for this: One important attribute is the expression level of each gene, another attribute is the extent of similarity to other sequences in the dataset.

A higher expression level typically is associated with a wider range of optimal assembly parameters (Figure 2). Not only does the expression level affect the range of coverage cutoffs but also the range of k-mer sizes. However, if a gene has a very high expression level, as with ESM1 and rbcS in *P*. *fastigiatum*, this effect seems to be reversed. The reads for these two transcripts can be assembled fairly well when separated from the rest of the dataset, especially in the case of ESM1. However, even the addition of only the reads with up to three mismatches does cause a fragmented assembly. This is surprising because our experience is that allowing for mismatches with less highly expressed genes tends to reduce fragmentation. Combining the reads of the seven example sequences generated an extremely fragmented assembly for these two transcripts resulting in very short sequences (< 120 bp). Since contigs smaller than 100 or 200 bp are normally excluded from further analyses as they are too short to be accurately annotated, contigs of very highly expressed genes will be absent from assemblies made with low coverage cutoffs (thus it will appear as if they were not expressed at all). Both ESM1 and rbcS belong to gene families with highly similar paralogous sequences [34,35]. The presence of these might provide an explanation for the fragmented assemblies obtained with these genes.

The three gene copies for MVP1 are highly similar and thus require assembly using higher k-mer values. However the transcripts for these copies have a low to medium expression level, which means that high k-mer values are not appropriate. A tradeoff seems to be k-mer sizes 51 and 53 with which all sequences can be assembled to nearly full length transcripts.

Assembly of the transcripts for rbcL and AT1G75680 required accommodating low levels of gene expression. In this situation contigs might not be joined because there are too few reads connecting them. Including reads with mismatches in this instance is expected to help the assembly as the presence of these can increase read coverage. This was found to be the case in the assembly of rbcL. This gene is chloroplast encoded, and therefore only one copy of this gene exists, thus there were no reads stemming from a similar homeologous or paralogous copy to interfere with the assembly. This also seems to have been the case with AT1G75680. That is, while it is nuclear located, the inclusion of mismatched reads improved the assembly regardless of the k-mer size and cutoff values. This hints either towards the presence of only a very dissimilar homeologous copy in the transcriptome or towards a lack of expression of a homeologous copy.

In summary, our observations suggest that common causes of fragmented assemblies with allopolyploid libraries are either too low or too high expression levels and a high degree of similarity between homeologous or paralogous sequences. All of these issues can be addressed using different strategies, however the success of these strategies depends on multiple features of the genes. For example, the inclusion of mismatched reads can assist in the assembly of transcripts with a low expression level. However, the addition of mismatched reads can introduce significant noise into a dataset of reads when transcripts are highly expressed. In this instance their inclusion will be unhelpful.

## Conclusions

As many gene families have arisen through gene duplication during evolution the similarity between different gene copies can pose a problem of significance in the assembly of transcriptomes. This is especially the case for organisms of polyploidy origin. The problem for assembly that highly similar gene copies cause can to some degree be overcome by altering the k-mer size and the coverage cutoff as shown here. While the addition of longer reads into the assemblies can also be expected to provide a solution for these cases, our findings highlight the potential of using even very short reads for the assembly of allopolyploid plant transcriptomes.

## Methods

### Plant growth

For the paired-end sequencing two *P*. *fastigiatum *accessions were grown from seed in a greenhouse at Landcare Research Lincoln. Seeds originated from plants collected at Ohau ski field (lane 3) and Serpentine creek (lane 4). Tissue samples from young and old leafs as well as roots were harvested and shock frozen in liquid nitrogen.

For single-end sequencing, seeds of *P*. *fastigiatum *(accession 'Serpentine creek') and *P*. *cheesemannii *(accession 'Kingston') were germinated according to [49] and young plants were transferred to potting mix (Orderings Nurseries Potting Mix). Leaves of three biological replicates per species were harvested and shock frozen in liquid nitrogen after cultivating the plants for 5 months in growth cabinets (Contherm Phytotron Climate Simulator) using the following parameters: 20°C, 50% air humidity, 180 uE PAR (photosynthetically active radiation), and 16 h day light.

### RNA extraction and sequencing

Total RNA for each sample was extracted using the RNeasy plant mini kit (QIAGEN, Hilden, Germany).

For the paired-end sequencing, RNA from young and old leaves was pooled in equal amounts before sample preparation for mRNA sequencing. Next, the combined leaf RNA and the root RNA from both *P*. *fastigiatum *accessions ('Ohau ski field' and 'Serpentine creek') underwent sample preparation using the mRNA-Seq sample prep Kit (Illumina, San Diego, CA USA) according to the manufacturer's instruction. For the 'Ohau' accession, leaves and roots were indexed with indices 1 and 2, respectively. For the 'Serpentine' accession, leaves and roots were indexed with indices 4 and 5, respectively. Indexed samples for each accession were pooled in equal molarity and loaded onto an Illumina flow cell (Flow cell number: 30173AAXX) at a concentration of 13.5 pmol per lane in lane 3 ('Ohau') and lane 4 ('Serpentine'). Both lanes were sequenced on a Genome Analyzer IIx (Illumina) for 75 cycles. The raw reads were uploaded to the NCBI SRA database under the accession numbers SRR364066 and SRR364067.

For the single-end sequencing, the three replicate *P*. *fastigiatum *(accession 'Serpentine creek') and *P*. *cheesemanii *(accession 'Kingston') RNA samples underwent separate sample preparation, using the mRNA-Seq sample prep Kit (Illumina, San Diego, CA USA) according to the manufacturer's instruction. The indexed samples for each accession were pooled in equal molarity and loaded onto an Illumina flow cell (Flow cell number: 437WUAAXX) at a concentration of 10.5 pmol per lane in lane 6 (*P*. *fastigiatum*) and lane 7 (*P*. *cheesemanii*). Both lanes were sequenced on a Genome Analyzer IIx (Illumina) for 75 cycles. The raw reads were uploaded to the NCBI SRA database under the accession numbers SRR364068, SRR364069, and SRR364070 for *P*. *fastigiatum *and SRR364073, SRR364072, and SRR364071 for *P*. *cheesemanii*.

### Quality assessment

The quality for each of the Illumina GAIIx 75 bp reads in all eight datasets from *P*. *fastigiatum *and *P*. *cheesemanii *was assessed using the program DynamicTrim [50] with a conservative threshold of 20 (Phred quality score), which is equivalent to one base call error every 100 nucleotides.. All reads that had less than 30 bp after trimming were discarded. Mates from paired-end reads, where one read was lost due to this filtering process, were considered single-end.

### *De novo* assembly

The reads for each species were assembled using ABySS v. 1.2.5 [25,26] using coverage cutoffs between two and 20. The k-mer length for each coverage cutoff was varied between 25 and 63 resulting in 380 different assemblies per species. Only contigs that were longer than 100 bp were retained. Sequences longer then 200 bp assembled with k-mer size 41 and coverage cutoff 7 for *P*. *fastigiatum *as well as 41 and five for *P*. *cheesemanii *were uploaded to the NCBI TSA (JP994808 - JP999999; JR000001 - JR034947; JP965884 - JP994807). All assemblies are available upon request.

For both species, the contigs from all 380 assemblies each were first analyzed separately. The number of contigs for each k-mer and coverage cutoff was determined as well as the number of sequences that were longer than 1000, between 500 and 1000, between 200 and 500, and between 100 and 200 nucleotides (for details see Additional file 1: Table S1). The respective longest sequence was extracted and annotated using BLAST [31] and the coding sequences of the TAIR10 database [51] and the JGI release v1.0 of the *Arabidopsis lyrata *genome [52]. BLAT [47] was used to search the contigs of each assembly against a combined database of coding sequences from *A*. *thaliana *and *A*. *lyrata *using an identity cutoff of 80%. For each contig only the longest hit in the reference library was retained and the percentage of the reference sequence covered was determined using a Perl script. Contigs that covered at least 95% of the reference coding sequence were considered as 'complete transcripts' and used for the further assessment of the assemblies. For each complete transcript, all assemblies in which this sequence could be found were determined using a Perl script.

All contigs that were identified as homologues to the same reference sequence (or its homologue in the respective other reference species) and covered more than 55% of that sequence were pooled together and further assembled using the overlap assembler CAP3 [32] with 98% overlap identity and 40 bp overlap. If the number of assembled supercontigs per coding sequence was greater than two, these sequences were analyzed separately as these sequences can either represent chimeric sequences between the two homeologous copies or a recent duplication of this gene that is not present in the reference library. The supercontigs were again compared to the reference sequences and the percentage of the reference sequence that was covered by this contig was determined. All sequences that covered at least 55% of the reference sequence were annotated according to the best BLAT hit in the reference database.

### Assessment of the assemblies

The sequences of both libraries were compared to the sequences of the same library to identify potential homeologous sequences as well as to the respective other library in order to identify orthologues using BLAST [31]. Those transcripts where four sequences could be identified, representing two homeologous transcripts in each species, were used to compute the minimal, mean and maximal amount of identity between the homeologues and between the orthologues. The remaining sequences were annotated according to these values.

Those contigs that spanned at least 95% of a reference sequence were extracted from the assemblies. BLAST was then used to determine the amount of overlap between the complete transcripts of two assemblies using an identity cutoff of 100%. The number of identical sequences between these datasets was determined using a Perl script and divided by the sum of number of complete transcripts in both datasets. A perfect overlap of two datasets resulted in a value of 0.5. These values were then divided by 0.5 to regain easily comparable percent values.

### Gene expression levels

The expression level of the completely assembled genes was derived by mapping all reads to the sequences of these genes and normalizing this value using following formula for each gene X [30]:

$$RPKM_{X}=1,000,000*\frac{M_{X}}{M_{A}*L_{X}}$$

where M_X _is the number of reads that mapped to the sequence of gene X, M_A _is the number of all reads mapping to all sequences, and L_X _is the length in kilobases of the sequence of gene X.

All statistical tests were conducted using R version 2.12.2 [53].

### Identification of best BLAST hits

The sequences that were longer than 100 bp of all 380 assemblies of *P*. *fastigiatum *were searched against a database containing all plant sequences in the refseq database (version 11.03.2011) using Blastx requesting a maximum of five target sequences.

## Abbreviations

BLAST: Basic local alignment search tool; NCBI: National center for biotechnology information; Nr: Non-redundant protein databank; TAIR: The *Arabidopsis *information resource

## Competing interests

The authors declare that they have no competing interests.

## Authors' contributions

NG assembled the data, analyzed the assemblies, designed the figures and drafted the manuscript. CV provided the plant material, extracted the RNA, and participated in drafting the manuscript. OD participated in the assembly of the data, and participated in drafting the manuscript. PL participated in the design of the study, and in drafting the manuscript. MB participated in drafting the manuscript, and provided the plant material. CE conducted the BLAST searches for the contigs of *P*. *fastigiatum *and participated in drafting the manuscript. All authors read and approved the final manuscript.

## Supplementary Material

Additional file 1 **Table S1. Number and length of contigs per k-mer size and coverage cutoff for *P*. *fastigiatum *and *P*. *cheesemanii***. The number of sequences obtained per coverage cutoff were counted and divided into four size classes: a) Sequences that were longer than 1000 bp, b) sequences shorter than 1000 bp but longer than 500 bp, c) sequences shorter than 500 but longer than 200 bp and d) sequences shorter than 200 but longer than 100 bp. Click here for file

Additional file 2 **Table S2. Statistics and longest sequences for 380 assemblies of the *P*. *fastigiatum *library**. The longest sequence was identified in each of the 380 assemblies and annotated according to its homologue in *A*. *thaliana*. The N50 and N90 values for each assembly were computed as well. Click here for file

Additional file 3 **Figure S1. Number of complete transcripts identified in different assemblies of *P*. *cheesemanii *reads**. 380 different assemblies were conducted using ABySS [25,26] and a combination of i) coverage cutoffs between 2 and 20 and II) k-mer sizes between 25 and 63. Transcripts covering the complete coding sequence of the homologue from *A*. *lyrata *or *A*. *thaliana *were identified and counted. The maximum number (558) of complete transcripts was identified for coverage cutoff five and k-mer size 41 while the lowest (58) number of complete transcripts was identified for coverage cutoff 19 and k-mer size 63. Click here for file

Additional file 4 **Supplementary file S1: 3,912 complete annotated transcripts from the library of *P*. *fastigiatum***. Contigs from all 380 assemblies of the *P*. *fastigiatum *reads were searched against a combined library of *A*. *lyrata *and *A*. *thaliana *coding sequences using BLAT [47]. Transcripts that spanned more than 55% of a reference coding sequence were extracted and, if none of the contigs allocated to a specific coding sequence spanned the whole sequence, assembled further with CAP3 [32]. All 3,912 transcripts that, after this step, spanned more than 95% of the reference coding sequence were considered as 'complete transcripts' and were annotated according to the *A*. *thaliana *TAIR accession number if possible and according to the *A*. *lyrata *transcript number elsewise. Click here for file

Additional file 5 **Supplementary file S2: 2,442 complete annotated transcripts from the library of *P*. *cheesemanii***. Contigs from all 380 assemblies of the *P*. *cheesemanii *reads were searched against a combined library of *A*. *lyrata *and *A*. *thaliana *coding sequences using BLAT [47]. Transcripts that spanned more than 55% of a reference coding sequence were extracted and, if none of the contigs allocated to a specific coding sequence spanned the whole sequence, assembled further with CAP3 [32]. All 2,442 transcripts that, after this step, spanned more than 95% of the reference coding sequence were considered as 'complete transcripts' and were annotated according to the *A*. *thaliana *TAIR accession number if possible and according to the *A*. *lyrata *transcript number elsewise. Click here for file

Additional file 6 **Table S3. Coverage cutoff values for seven genes made from assemblies of reads which had up to three mismatches**. All reads in the *P*. *fastigiatum *dataset mapping with up to three mismatches to the sequences of AT1G67090, AT3G14210, three sequences of AT1G54030 (two homeologues and one paralogue), ATCG00490, and AT1G75680 were determined and assembled separately using ABySS [25,26] and k-mer sizes 25 to 63. The automatically chosen mean coverage cutoff for each of the assemblies was extracted from the log files. Click here for file
