# Supplementary figures and images for: Cutoffs and k-mers: implications from a transcriptome study in allopolyploid plants
Source: BMC Genomics. 2012 Mar 14;13:92. doi: 10.1186/1471-2164-13-92 (PMC3378427; doi:10.1186/1471-2164-13-92)

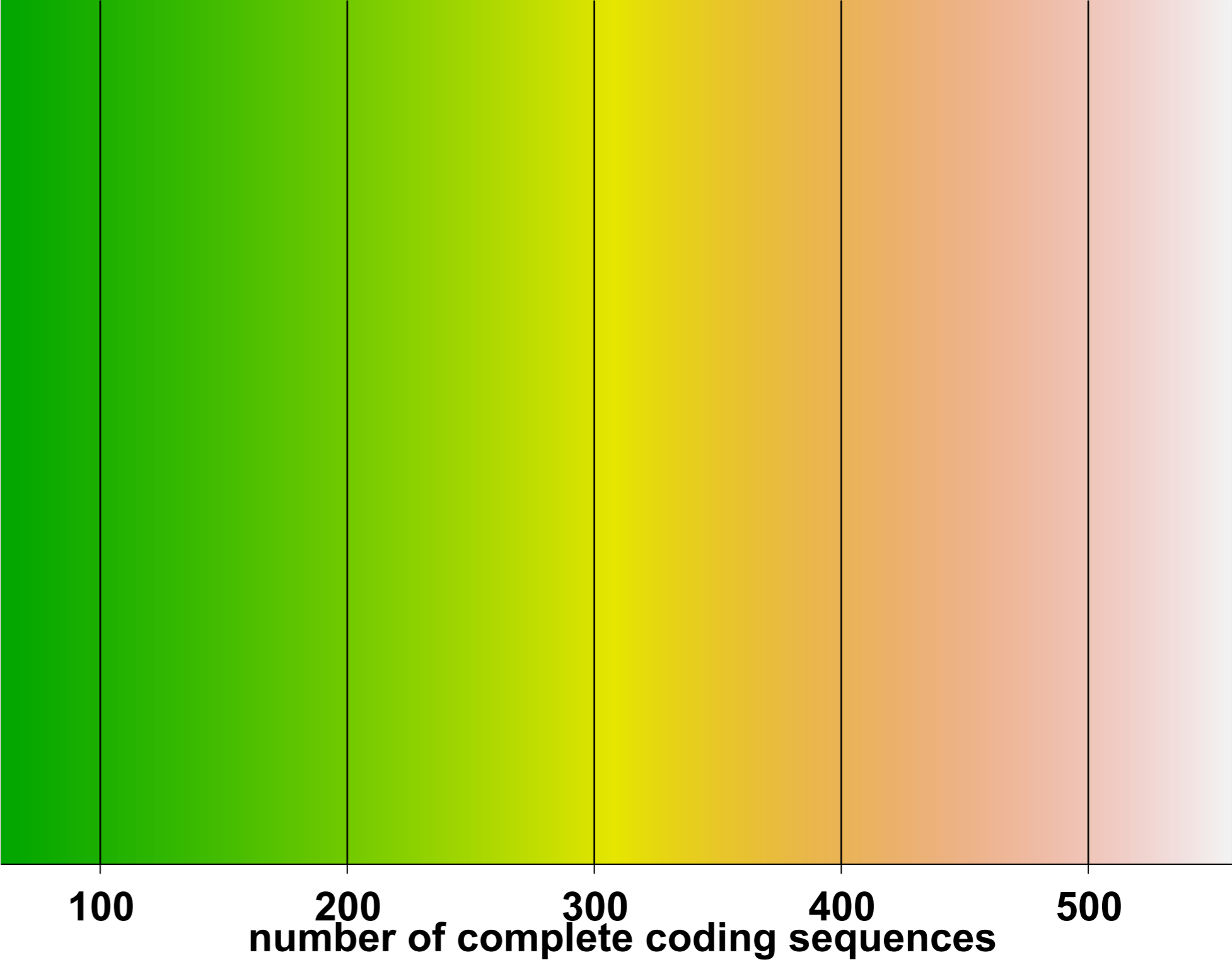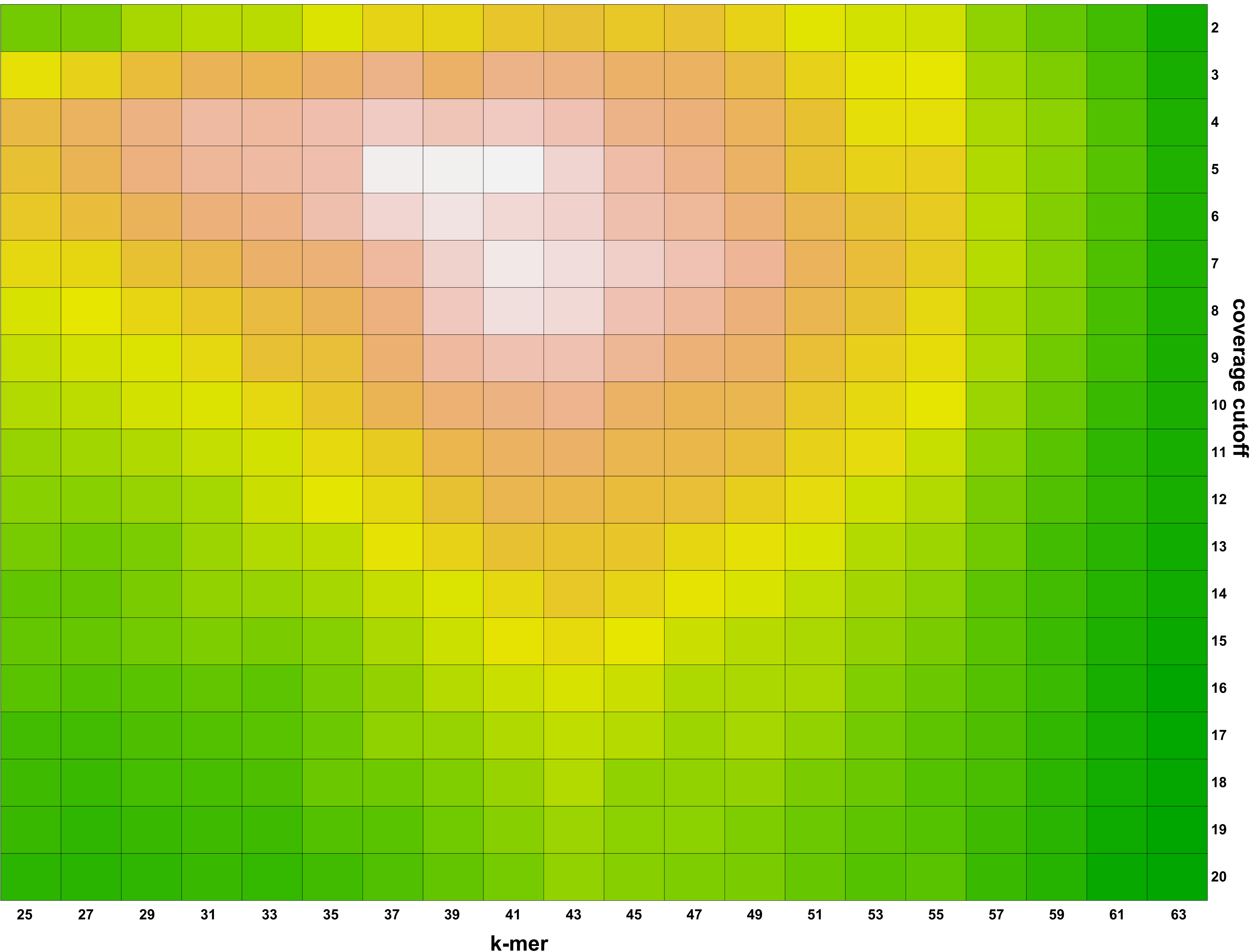

Supplement: Additional file 3 — Figure S1. Number of complete transcripts identified in different assemblies of P. cheesemanii reads. 380 different assemblies were conducted using ABySS [25,26] and a combination of i) coverage cutoffs between 2 and 20 and II) k-mer sizes between 25 and 63. Transcripts covering the complete coding sequence of the homologue from A. lyrata or A. thaliana were identified and counted. The maximum number (558) of complete transcripts was identified for coverage cutoff five and k-mer size 41 while the lowest (58) number of complete transcripts was identified for coverage cutoff 19 and k-mer size 63. [file 1471-2164-13-92-S3.PDF]
